# Preconceptual Zika virus asymptomatic infection protects against secondary prenatal infection

**DOI:** 10.1371/journal.ppat.1006684

**Published:** 2017-11-16

**Authors:** Lucien H. Turner, Jeremy M. Kinder, Adrienne Wilburn, Rahul J. D’Mello, Makayla R. Braunlin, Tony T. Jiang, Giang Pham, Sing Sing Way

**Affiliations:** 1 Division of Infectious Diseases and Perinatal Institute, Cincinnati Children’s Hospital, Cincinnati, Ohio, United States of America; 2 Immunology Graduate Program, Cincinnati Children’s Hospital Medical Center and University of Cincinnati College of Medicine, Cincinnati, Ohio, United States of America; NIH, UNITED STATES

## Abstract

Pregnant women, and their fetal offspring, are uniquely susceptible to Zika virus and other microbial pathogens capable of congenital fetal infection. Unavoidable exposure to Zika virus in endemic areas underscores the need for identifying at-risk individuals, and protecting expecting mothers and their fetal offspring against prenatal infection. Here we show that primary Zika virus asymptomatic infection in mice confers protection against re-infection, and that these protective benefits are maintained during pregnancy. Zika virus recovery was sharply reduced in maternal tissues and amongst fetal concepti after prenatal challenge in mothers with resolved subclinical infection prior to pregnancy compared with mice undergoing primary prenatal infection. These benefits coincide with expanded accumulation of viral-specific antibodies in maternal serum and fetal tissues that protect against infection by the identical or heterologous Zika virus genotype strains. Thus, preconceptual infection primes Zika virus-specific antibodies that confer cross-genotype protection against re-infection during pregnancy.

## Author summary

Expecting mothers are uniquely susceptible to Zika virus infection that often spreads to vital tissues of the developing fetus. Since Zika virus infection in healthy non-pregnant individuals is mostly asymptomatic, a large proportion of reproductive age women that live in Zika endemic areas have been previously infected, and cleared the infection prior to pregnancy. Here we show that primary Zika virus asymptomatic infection in mice protects against re-infection, and that these protective benefits are maintained during pregnancy. Protection in this preclinical model is mediated by circulating antibodies found at very high levels amongst individuals with resolved infection that efficiently neutralize virus infectivity. Thus, antibodies against Zika virus, naturally stimulated by prior asymptomatic infection, protect against re-infection during pregnancy, and the presence of these protective antibodies may help discriminate protected individuals from others that remain susceptible to infection. This knowledge, combined with protection primed by promising candidate vaccine formulations that also stimulate production of high level neutralizing antibodies, will help identify reproductive age women at-risk for severe infection consequences and more focused therapeutic strategies for averting infection.

## Introduction

The ongoing Zika virus (ZIKV) epidemic has triggered an explosion in cases of fetal death, microcephaly and other birth defects in surviving infants with congenital infection [1–4]. These sequelae usually occur in parallel with higher and more prolonged maternal viremia for up to 15 weeks following prenatal infection [5–9]. By contrast, ZIKV infection in non-pregnant individuals is mostly subclinical or asymptomatic, and associated with only transient self-resolving viremia [10]. For example, only 19% of ZIKV IgM seropositive individuals reported clinical symptoms during the 2007 Yap Island outbreak [11]. Likewise, only 11% of individuals developed reportable symptoms despite an approximate 50% rate of newly acquired seroprevalance during the 2013–2014 French Polynesian outbreak [12]. Thus, considering ZIKV-associated morbidity is largely confined to infection during pregnancy, there is an urgent need for improved strategies for protecting against congenital fetal invasion and prenatal infection susceptibility. This urgency persists even though several ZIKV candidate vaccines have recently been shown to confer very promising protection in animal infection models [13–21], since clinical validation of efficacy and safety, especially with increasingly recognized immunological shifts that physiologically occur during human allogeneic pregnancies, have not been demonstrated.

Given the limited and non-specific clinical symptoms associated with most ZIKV infections in healthy non-pregnant individuals, a fundamental unanswered question regarding prenatal ZIKV susceptibility is whether preconceptual infection protects against re-infection during pregnancy. For some classical prenatal pathogens (e.g. varicella virus, rubella virus), maternal susceptibility and congenital fetal invasion are each efficiently overturned amongst women with resolved infection prior to pregnancy [22–24]. By contrast, protection conferred by preconceptual infection is considerably less reliable for other prenatal pathogens—that may reflect susceptibility to re-infection by immunologically discordant strains (e.g. human cytomegalovirus, influenza virus) [25, 26], or attenuated responsiveness of normally protective maternal immune components simultaneously required for sustaining fetal tolerance (e.g. *Plasmodium* spp., *Listeria monocytogenes*) [27–29]. Importantly, while prior infection has been shown to protect against lethal re-challenge in non-pregnant hosts for other flaviviruses such as West Nile virus (WNV) or St. Louis encephalitis virus [30–32], whether primary infection protects against re-infection during pregnancy for flaviviruses as a group remains poorly defined since prenatal infection has not traditionally been a dominant feature for these viral pathogens until the recent clinical emergence of ZIKV [33].

Herein, pregnancies established among genetically discordant strains of inbred mice were used to investigate how primary preconceptual ZIKV infection impacts the susceptibility of mothers and their fetal offspring to re-infection during pregnancy. We show that anti-viral immunity primed by asymptomatic infection prior to pregnancy protects against re-infection during pregnancy, with sharply reduced susceptibility in both maternal and fetal tissues. These findings have important translational implications for discriminating individuals at risk for severe prenatal infection from others with naturally acquired protective immunity, and new strategies for protecting against congenital fetal invasion.

## Results

### Primary ZIKV asymptomatic infection protects against re-infection

**Fig 1:**
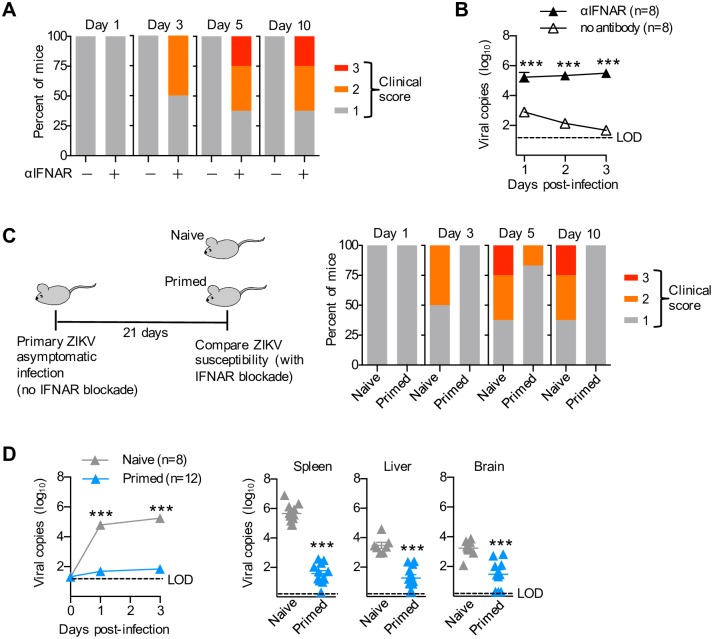
Asymptomatic ZIKV primary infection protects against re-infection. (**A**) Bar graph comparing the incidence and clinical symptom severity (clinical score) among mice administered anti-type I IFN receptor antibody (n = 8) beginning one day prior to infection (10^6^ PFUs PRVABC59) compared with no antibody controls (n = 15) pooled from three independent experiments each with similar results. (**B**) ZIKV genome copies per mL serum for mice described in panel A. (**C**) Schematic illustrating re-infection amongst mice with prior asymptomatic infection (primed, n = 12) compared to primary infection in naive control mice (n = 12); and bar graph comparing the incidence and clinical symptom severity (clinical score) after ZIKV infection with type I IFN receptor blockade among each group pooled from three independent experiments each with similar results. (**D**) ZIKV genome copies per mL serum and in each tissue day 3 after infection for mice described in panel C. Each point depicts the data from an individual mouse that is representative of at least three independent experiments each with similar results. Bar, mean ± one standard error; LOD, limits of detection; *** *p* < 0.001.

ZIKV shares with other flaviviruses, susceptibility to innate anti-viral immunity activated by type I interferons (IFNs), and functionally overriding host cell type I IFN responsiveness is required for productive symptomatic infection [34, 35]. We exploited the selective resistance of murine cells to STAT2 degradation by ZIKV NS5 protein, reasoning that the natural innate resistance from unabated type I IFN responsiveness in mice makes this species ideally suited to probe immunological shifts primed by asymptomatic infection in non-pregnant individuals [36, 37]. In turn, temporally overriding innate protection by initiating type I IFN receptor antibody blockade immediately prior to secondary challenge creates a unique opportunity for investigating the impacts of prior asymptomatic infection on susceptibility to re-infection. Consistent with the results of recent studies [34, 35], standard laboratory C57BL/6 mice inoculated with a ZIKV clinical isolate (PRVABC59) from the contemporary Latin American outbreak showed no clinical symptoms and rapidly cleared the infection (Fig 1A and 1B). Comparatively, clinical signs of infection (i.e., hunched posture, ruffled hair, lethargy) that paralleled high levels of circulating virus were unleashed by type I IFN receptor antibody (MAR1-5A3) blockade administered beginning one day prior to infection (Fig 1A and 1B). Thus, antibody mediated type I IFN receptor blockade efficiently causes clinical and virological susceptibility to ZIKV infection despite previously described normal weight gain in mice [34].

To investigate how prior infection impacts susceptibility to re-infection, clinical symptoms and viral RNA levels after secondary ZIKV challenge amongst mice with prior asymptomatic infection were compared with primary infection in naive control animals. Interestingly, despite susceptibility conferred by type I IFN receptor blockade initiated one-day prior to infection, clinical symptoms were sharply attenuated amongst mice with prior asymptomatic infection compared with naive control mice (Fig 1C). These protective benefits paralleled significantly reduced recovery of ZIKV RNA in the blood and tissues of mice undergoing secondary challenge compared with primary infection in naive controls (Fig 1D). Together with recent studies demonstrating significantly reduced susceptibility to secondary compared with primary ZIKV infection in non-human primates [38–40], these results highlight the immunogenicity of ZIKV where even abortive primary infection can efficiently prime protection against re-infection.

### Neutralizing IgG antibodies primed by primary ZIKV asymptomatic infection protect against re-infection

A variety of adaptive immune components stimulated by ZIKV infection or candidate vaccine formulations are associated with protection. For example, polyfunctional IFN-γ plus TNF-α producing CD8^+^ T cells with broad ZIKV specificity primed by primary infection can overturn the susceptibility of naive recipient mice after adoptive transfer *in vivo* [41, 42]. Likewise, sterilizing immunity induced by live attenuated or inactivated ZIKV candidate vaccine formulations occurs in parallel with sharply expanded accumulation of IFN-γ producing ZIKV-specific CD4^+^ and CD4^+^ T cells [14, 15]. On the other hand, the same live attenuated viral strains and nucleic acid based candidate vaccines each prime high titer ZIKV-specific IgG antibodies that protect against infection in non-pregnant mice and non-human primates [13–18]. ZIKV susceptibility to antibodies is further highlighted by protection against infection in pregnant and non-pregnant mice adoptively transferred individual clones of human monoclonal antibodies that bind and neutralize ZIKV in vitro infectivity most efficiently [43]. Thus, while ZIKV-specific T cells and antibodies are each capable of protection, the adaptive immune components stimulated by subclinical infection that protect against re-infection remain undefined. Accordingly, our model of protective immunity primed by primary ZIKV asymptomatic infection was used to evaluate the relative contribution of serological compared with cellular adaptive immune components.

**Fig 2:**
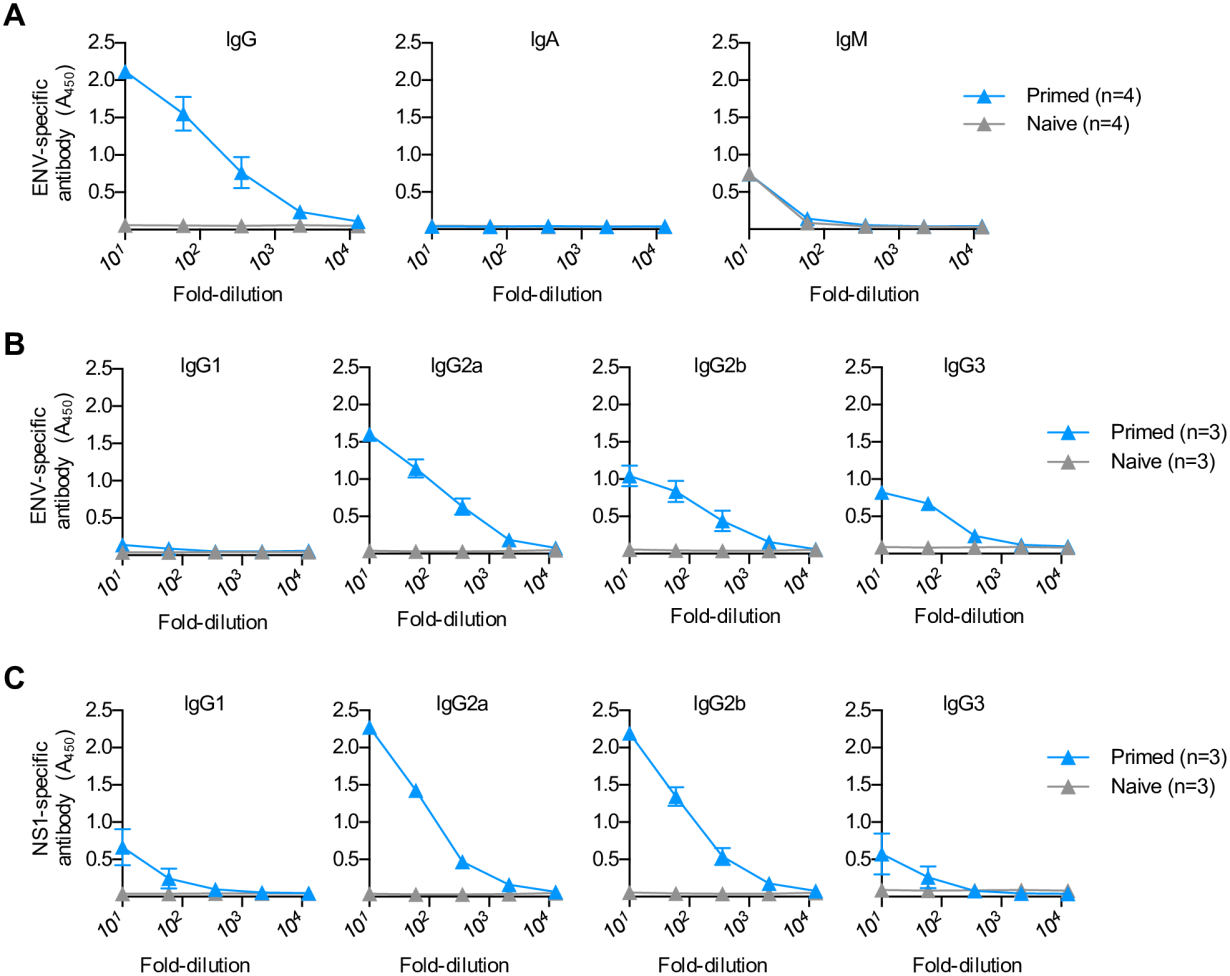
Primary ZIKV asymptomatic infection primes expanded accumulation of IgG antibodies. (**A**) IgG, IgA and IgM antibody titers (A_450_) with ZIKV ENV specificity in the serum of mice day 21 after asymptomatic primary infection compared with naive control mice. (**B**) IgG1, IgG2a, IgG2b, and IgG3 antibody titers (A_450_) with ZIKV ENV specificity in the serum of mice described in panel A. (**C**) IgG1, IgG2a, IgG2b, and IgG3 antibody titers (A_450_) with ZIKV NS1 specificity in the serum of mice described in panel A. These data are representative of at least three independent experiments each with similar results. Bar, mean ± one standard error.

**Fig 3:**
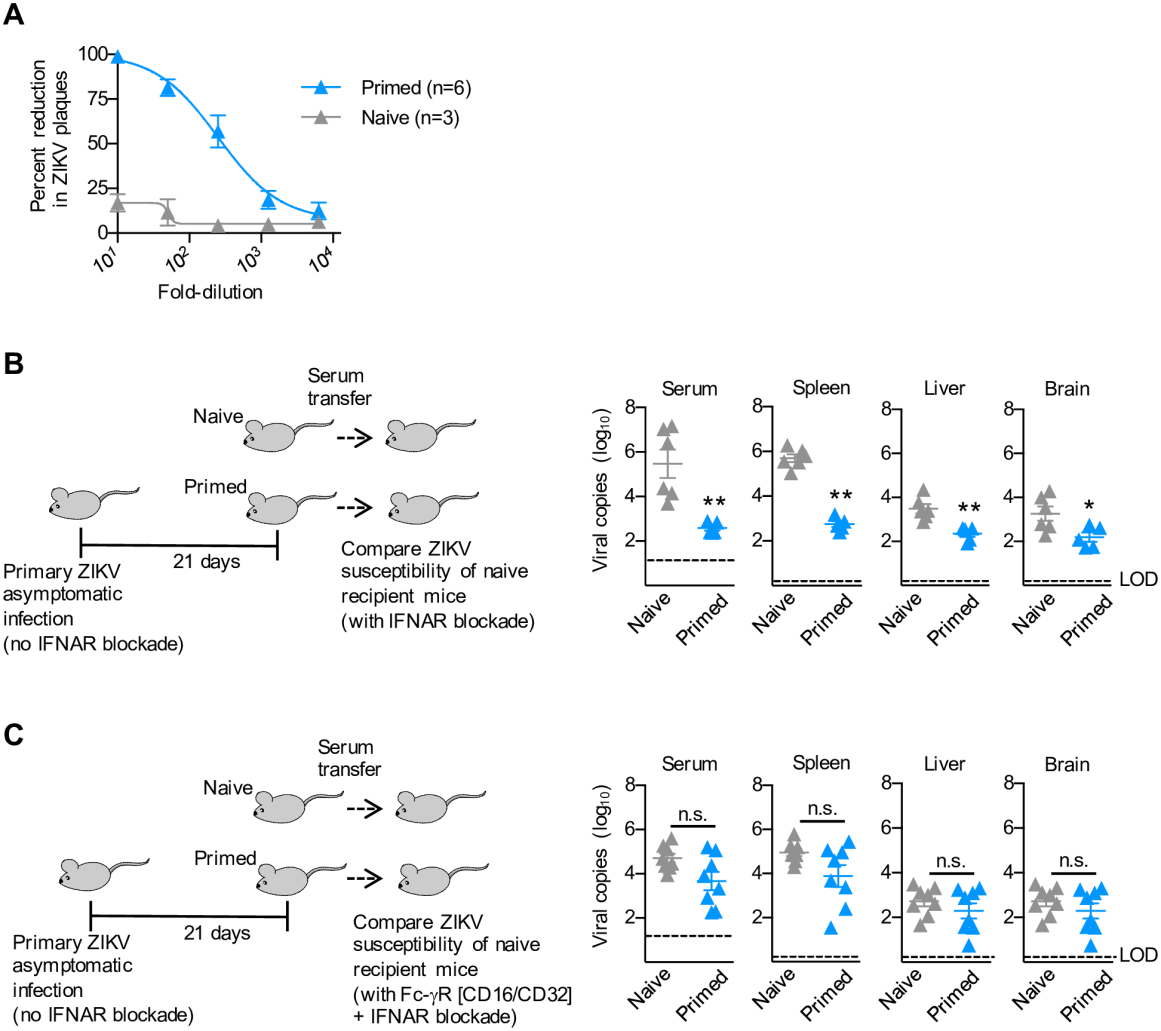
Primary ZIKV asymptomatic infection primes neutralizing antibodies that protect against re-infection. (**A**) Percent reduction in ZIKV plaques after pre-incubation with each dilution of the serum of mice day 21 after asymptomatic primary infection compared with naive control mice. (**B**) Schematic comparing the susceptibility of naive recipient mice administered serum from donor mice with prior asymptomatic infection or naive control mice; and ZIKV genome copies in the serum or each tissue day 3 after infection. (**C**) Schematic comparing the susceptibility of naive recipient mice administered Fcγ receptor neutralizing antibody, along with serum from donor mice with prior asymptomatic infection or naive control mice; and ZIKV genome copies in the serum or each tissue day 3 after infection. Each point depicts the data from an individual mouse that is representative of at least three independent experiments each with similar results. Bar, mean ± one standard error; LOD, limits of detection; * *p* < 0.05; ** *p* < 0.01; *** *p* < 0.001; n.s. not significant.

Consistent with efficient serological conversion after human subclinical infection [11, 12, 44], the serum of mice three weeks after asymptomatic primary infection showed robust accumulation of ZIKV-specific IgG antibody, whereas IgA and IgM titers remained at background levels found in naive control mice (Fig 2A). ZIKV-specific IgG antibodies were highly enriched for IgG2a and IgG2b subclasses, with specificity to ZIKV envelope (ENV) and NS1 proteins (Fig 2B and 2C). In turn, serum from mice three weeks after primary asymptomatic infection also efficiently neutralized ZIKV plaque formation in Vero cell monolayers, with serial dilutions of the serum showing reductions in functional activity that directly paralleled when the titer of virus-specific IgG antibody returned to background levels (loss of activity for both after 10^3^ to 10^4^-fold dilutions) (Fig 3A compared with Fig 2A). Interestingly, heat-inactivation did not significantly impact the neutralization potency of serum from mice with prior asymptomatic infection suggesting heat-liable serum components (e.g. complement) are not required for neutralizing ZIKV *in vitro* infectivity (S1A Fig).

To further investigate whether ZIKV-specific antibodies primed by asymptomatic primary infection protect against infection, the susceptibility of naive mice adoptively transferred serum from donor mice infected with ZIKV three weeks prior was evaluated. ZIKV RNA levels were significantly reduced in the serum, spleen, liver and brain of recipient mice prophylactically treated one day prior to infection with serum from donor mice with resolved primary asymptomatic infection compared with the serum of naive control mice (Fig 3B). Interestingly, Fc-γ receptor (CD16/CD32) *in vivo* blockade [45, 46] efficiently overturned the protective benefits of adoptively transferred serum from mice with resolved primary infection, highlighting essential roles for phagocytic host cells that take up antibody coated virus through Fc-γ receptors (Fig 3C). In contrast, depletion of CD8^+^ or CD4^+^ T cells, either individually or in combination, did not significantly alter protection against re-infection amongst mice with prior infection (S1B Fig). Thus, protection against ZIKV re-infection primed by primary infection is associated with retained accumulation of high titer viral-specific antibodies that can adoptively transfer protection to naive recipients. Together with the protective benefits of exogenously administered ZIKV-specific IgG antibodies shown in recent studies [43, 47], these findings suggest high titer ZIKV antibodies that inhibit viral infectivity can bypass the necessity for CD8^+^ T cells during primary infection [41, 48].

### Preconceptual asymptomatic ZIKV infection protects against re-infection during pregnancy

**Fig 4:**
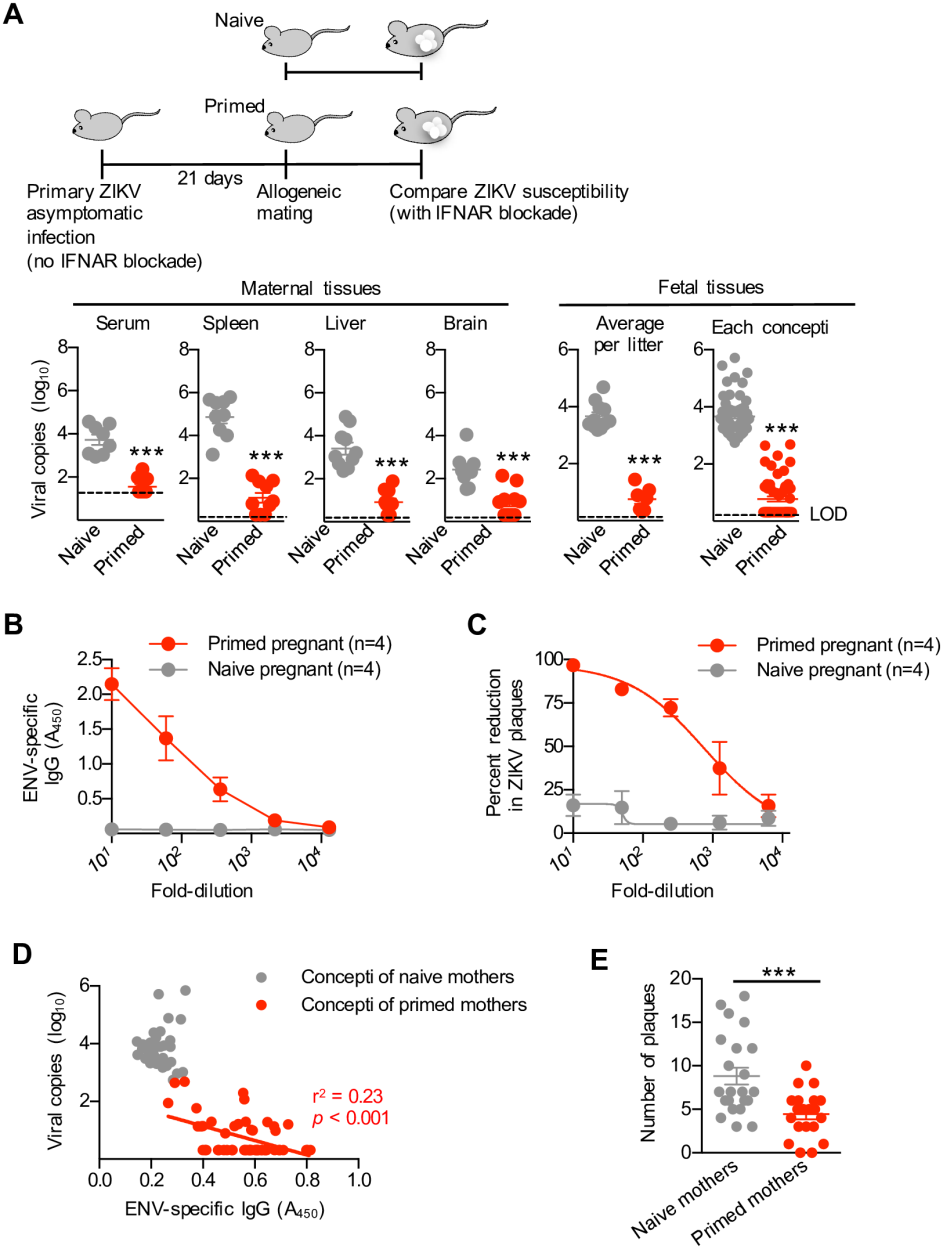
Primary ZIKV asymptomatic infection protects against re-infection during pregnancy. (**A**) Schematic illustrating when mating is initiated and secondary ZIKV infection occurs amongst mice with prior asymptomatic primary infection compared with naive control mice. ZIKV genome copies in each maternal tissue, each individual concepti (placenta, decidua, and fetal tissue), or averaged among individual concepti in each litter day 3 after ZIKV re-infection at midgestation (E11.5). (**B**) IgG antibody titers (A_450_) with ZIKV ENV specificity in the serum of mice described in panel A (**C**) Percent reduction in ZIKV plaques after pre-incubation with each dilution of the serum each group of mice described in panel A. (**D**) Scatterplot comparing ZIKV genomic copies and ENV IgG antibody levels (A_450_) in clarified fetal homogenates amongst individual concepti described in panel A. (**E**) Number of ZIKV plaques after pre-incubation with clarified homogenates of individual concepti described in panel A. Each point depicts the data from an individual mouse that is representative of at least three independent experiments each with similar results. Bar, mean ± one standard error; LOD, limits of detection; * *p* < 0.05; ** *p* < 0.01; *** *p* < 0.001.

Given the sharply increased morbidity associated with ZIKV infection during pregnancy—with ensuing congenital invasion of fetal tissues, we further investigated whether protection primed by preconceptual infection persists during pregnancy. Three weeks after primary asymptomatic infection, allogeneic pregnancies were established amongst C57BL/6 (H-2^b^) female mice by mating with Balb/c (H-2^d^) males to recapitulate the natural heterogeneity between maternal and fetal antigens in outbred populations. Type I IFN receptor blockade was subsequently initiated in pregnant females at midgestation (E10.5), followed by ZIKV infection one-day later (E11.5) (Fig 4A). Interestingly, despite immunological shifts required for averting fetal rejection, the presence of expanded fetal target tissue and diminished responsiveness of maternal T cells to primary ZIKV infection during pregnancy [49], protection against secondary challenge was maintained amongst mice with prior preconceptual infection shown by sharply reduced ZIKV RNA levels in their serum, spleen, liver and brain compared with primary prenatal infection in naive control mice (Fig 4A). Importantly, congenital invasion was also efficiently averted as ZIKV RNA levels were reduced to near or below the limits of detection for most concepti (fetal plus decidual tissue) after prenatal challenge of mice with prior preconceptual infection (Fig 4A).

Protection against re-infection during pregnancy paralleled persistence of high titer circulating ZIKV IgG antibodies that efficiently neutralized ZIKV plaque formation *in vitro* (loss of activity for both after 10^3^ to 10^4^-fold dilutions) following preconceptual primary infection compared with naive control mice (Fig 4B and 4C). Interestingly, despite high levels of neutralizing antibodies in maternal serum following preconceptual primary infection, sporadic, low level placental ZIKV dissemination occurred with secondary prenatal infection (Fig 4A). These findings are consistent with breakthrough congenital invasion in mice receiving preconceptual vaccination or exogenous ZIKV-antibody transfer prior to prenatal infection [20, 21, 43]. To investigate the possibility that vertically transferred maternal antibodies may provide additional protective benefits, the levels of ZIKV-specific antibodies were compared with viral RNA levels amongst individual concepti. ZIKV-specific IgG levels were sharply increased in nearly all concepti homogenates of pregnant mice with prior preconceptual infection compared with the concepti of naive control mice, with a highly significant inverse correlation with ZIKV RNA levels amongst individual concepti scattered across multiple litters with low level breakthrough infection (Fig 4D). Likewise, ZIKV infectivity of Vero cells was significantly reduced by pre-incubation with UV-inactivated fetal tissue homogenates of pregnant mice with prior preconceptual infection compared with the concepti of naive control mice (Fig 4E). Together with recent studies demonstrating protection against congenital ZIKV transmission primed by mRNA or live attenuated viral vaccine platforms [20, 21], or amongst mice exogenously administered ZIKV human monoclonal antibody with high neutralization potency [43], these findings highlight the protective capacity of virus-specific neutralizing antibodies in overturning the natural vulnerability of mothers and their fetal offspring to ZIKV prenatal infection.

### Asymptomatic primary ZIKV infection protects against re-infection by heterologous viral genotype strains

ZIKV is believed to have originated in East Africa, with subsequent mutation into unique West African and Asian variants [50]. Despite incomplete information on whether selective pressures by host immunity drive these antigenic shifts, the existence of unique ZIKV lineage strains has important practical implications for the scope of protection primed by natural infection or vaccination. For example, while purified ZIKV-specific human monoclonal antibodies can effectively neutralize both Asian and African strains, a single amino acid mutation in ZIKV ENV protein can override neutralization by individual antibody clones [43]. Furthermore, while antibodies primed by infection with the related flavivirus, Dengue (DENV), protect against secondary infection by identical viral serotype strains, they can also enhance susceptibility to re-infection by discordant DENV serotypes [51–53].

**Fig 5:**
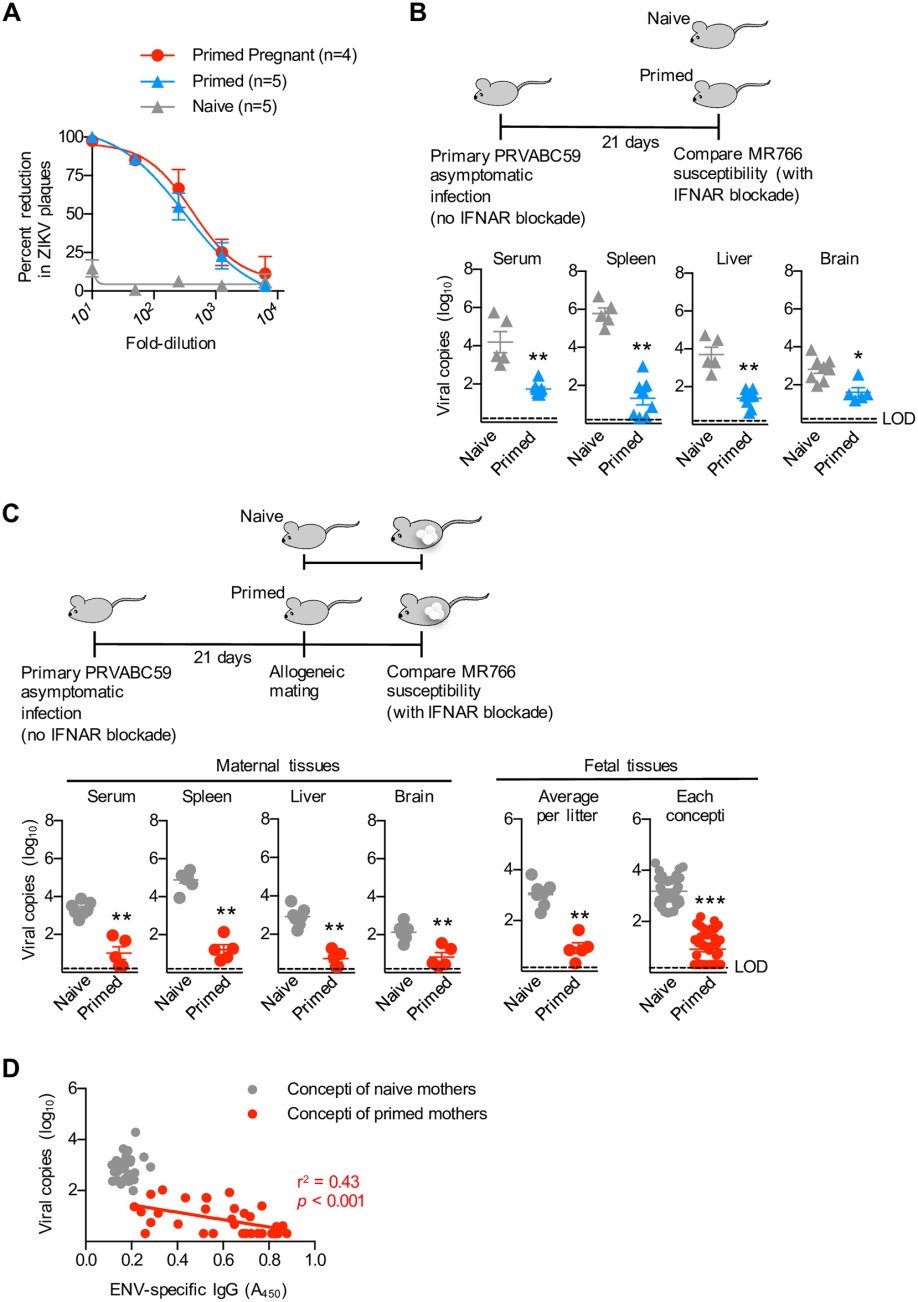
Primary ZIKV asymptomatic infection protects against re-infection by heterologous viral genotype strains. (**A**) Percent reduction in MR766 plaques after pre-incubation with each dilution of the serum from virgin or mid-gestation (E11.5) pregnant mice with prior asymptomatic primary PRVABC59 infection compared with the serum of naive control mice. (**B**) Schematic illustrating heterologous MR766 infection among type I IFN receptor neutralized mice with prior asymptomatic PRVABC59 infection compared with naive control mice; and ZIKV genome copies in the serum and each tissue day 3 after MR766 infection for each group of mice. (**C**) Schematic illustrating when mating is initiated and secondary MR766 ZIKV infection occurs amongst mice with asymptomatic primary PRVABC59 ZIKV infection compared with naive control mice. ZIKV MR766 genome copies in each maternal tissue, each individual concepti (placenta, decidua, and fetal tissue), or averaged among individual concepti in each litter day 3 after ZIKV re-infection at midgestation (E11.5). (**D**) Scatterplot comparing ZIKV MR766 genomic copies and ENV IgG antibody levels (A_450_) in clarified fetal homogenates amongst individual concepti for the mice described in panel C. Each point depicts the data from an individual mouse that is representative of at least three independent experiments each with similar results. Bar, mean ± one standard error; LOD, limits of detection; * *p* < 0.05; ** *p* < 0.01; *** *p* < 0.001.

To investigate whether protective immunity primed by primary ZIKV infection extends to cross-lineage ZIKV strains, susceptibility to the original Uganda East African MR766 strain primed by prior infection with the Asian lineage PRVABC59 strain used in our preceding experiments was evaluated. We found the serum from mice with prior PRVABC59 infection efficiently neutralized plaque formation by the cross-lineage MR766 strain, and with near identical potency compared with monolayers infected with the homologous PRVABC59 virus (loss of activity between 10^3^ to 10^4^-fold dilutions) (Fig 5A compared with Fig 3A). In turn, the potency of MR766 neutralization by the serum of mice with prior PRVABC59 infection was not significantly impacted by pregnancy (Fig 5A).

In agreement with this cross-lineage susceptibility of MR766 to antibodies primed by prior PRVABC59 infection, ZIKV RNA levels were significantly reduced after secondary MR766 challenge amongst mice with prior asymptomatic PRVABC59 infection compared with primary MR766 ZIKV infection in naive control mice (Fig 5B). Importantly, cross-lineage protection primed by preconceptual prior infection is maintained during pregnancy shown by significantly reduced ZIKV RNA in the maternal serum spleen, liver, and brain, and amongst individual concepti (fetal plus decidual tissue) after MR766 secondary challenge in midgestation pregnant mice with preconceptual PRVABC59 infection compared with primary MR766 prenatal infection in naive control mice (Fig 5C). Significantly increased ZIKV IgG antibody titers that were inversely associated with ZIKV RNA levels were found in the tissue homogenate of nearly all concepti recovered from protected pregnant mice with preconceptual primary infection, but absent in concepti from naive control mice (Fig 5D). Thus, the broadly neutralizing capacity of serum after primary ZIKV infection in humans, non-human primates and mice [38, 40, 54], extends to cross-genotype protection against re-infection during pregnancy.

## Discussion

**Fig 6:**
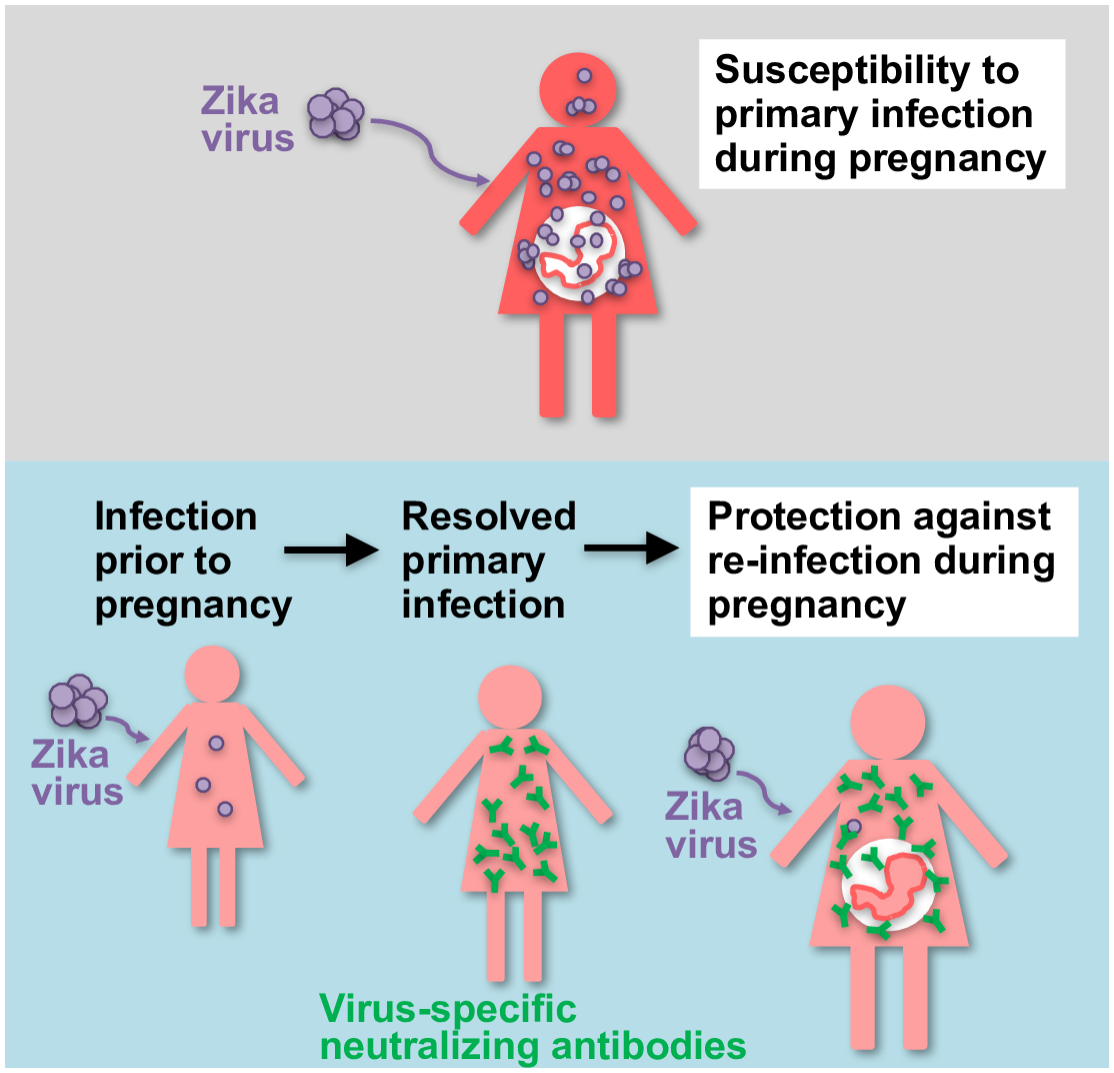
Preconceptual Zika virus infection protects against re-infection during pregnancy. Zika virus primary infection during pregnancy causes widespread seeding of maternal tissues and congenital fetal invasion (top), whereas asymptomatic ZIKV primary infection prior to pregnancy protects against re-infection during pregnancy (bottom).

Despite identification nearly 70 years ago, ZIKV has remained a relatively obscure human pathogen until its emergence and global spread beginning in 2015. The unique propensity for congenital fetal invasion makes ZIKV distinct from other flaviviruses, and opens-up many unanswered fundamental questions for ZIKV and other pathogens that cause prenatal infection. These include whether strategies that protect against infection in non-pregnant healthy individuals remain effective despite pregnancy-associated anatomical changes that significantly expands susceptible target tissue to include fetal tissues, and increasingly recognized immunological shifts that avert maternal-fetal immunological conflict and dampen the proliferation-activation of T cells after infection during pregnancy [49, 55, 56]. Here we show preconceptual asymptomatic ZIKV primary infection protects against re-infection, and that these protective benefits are maintained during pregnancy (Fig 6). Thus, naturally acquired immunity against prenatal ZIKV infection is similar to the resistance of mothers to classical prenatal pathogens (e.g. varicella virus, rubella virus) that is also efficiently primed by preconceptual infection. A potentially unifying theme among these pathogens is diversity of protective epitopes that functionally minimizes the impacts of antigenic shifts amongst individual immune dominant microbe expressed antigens. For naturally acquired immunity to ZIKV, this notion is supported by the wide distribution of protective epitopes across spatially distinct domains of immune dominant envelope protein [13–18, 43, 57–59]. By contrast, the number of protective immune dominant epitopes is more limited for other viral pathogens (e.g. human cytomegalovirus, influenza virus) where protection after preconceptual infection is less reliable [25, 26].

These protective benefits conferred by pre-conceptual asymptomatic infection parallel sustained accumulation of ZIKV-specific antibodies in maternal serum that efficiently neutralize virus infectivity *in vitro* and reduce susceptibility to *in vivo* infection by the identical or heterologous ZIKV genotype strains. Interestingly, serum from mice primed by pre-conceptual infection efficiently neutralized ZIKV infectivity despite prior heat-inactivation, suggesting these protective benefits do not require complement or other heat-liable components associated with enhanced protection for other flaviviruses such as WNV [60]. On the other hand, antibody-mediated Fcγ receptor blockade overturned protection conferred by transfer of serum from mice with prior preconceptual infection. These results are consistent with our finding that primary asymptomatic infection selectively primes IgG2a and IgG2b antibodies with ZIKV ENV and NS1 specificity, and high affinity Fcγ receptor binding for these specific mouse antibody isotypes [61, 62]. Interestingly however, inactivation of Fcγ receptor binding for a human monoclonal IgG1 antibody with high affinity for ZIKV ENV protein does not significantly impact protection against prenatal ZIKV infection in mice [43]. Thus, further studies are needed to investigate how the necessity for Fcγ receptor can be functionally bypassed, in particular focusing on the importance of antibody isotype and/or antigen affinity, and the potential for cross-reactivity of ZIKV-specific antibodies with structurally homologous flaviviruses such as DENV that may promote antibody-dependent enhanced infectivity [63, 64].

Complexities inherent to investigating immunity and the pathogenesis of prenatal infection largely stem from the choice of experimental models that need to balance practicality with relevance to human pregnancy. Here, it is important to highlight that only humans have human placentas—with anatomical and molecular features that are not reproduced in any other species [65]. Likewise, the physiological discordance between maternal and fetal-expressed paternal antigens drive potent immunological shifts during pregnancy amongst humans and other outbred species that convey profound impacts on prenatal infection susceptibility [66]. For example, expanded accumulation of immune suppressive regulatory CD4^+^ T cells is highly accentuated in allogeneic compared with syngeneic pregnancies amongst inbred strains of mice [67]. In turn, expanded systemic accumulation of immune suppressive regulatory CD4^+^ T cells promotes maternal susceptibility to common prenatal pathogens (e.g. *Listeria* and *Salmonella* spp.), whereas dampened suppressive function of maternal regulatory CD4^+^ T cells fractures fetal tolerance and promotes congenital invasion in the context of allogeneic pregnancy [67–69]. Therefore, to extend the analysis of ZIKV prenatal infection that in mice has been limited to syngeneic pregnancies, MHC haplotype discordant strains of inbred mice were used for breeding to recapitulate the physiological mis-match between maternal-fetal antigens encountered in human pregnancy. Strategies that render mice innately susceptible to more prolonged and higher levels of ZIKV viremia by administration of type I IFN receptor blocking antibodies were exploited to overcome the natural resistance of murine STAT2 to degradation by ZIKV NS5 protein [34–37]. We further reasoned that unabated type I IFN responsiveness that attenuates ZIKV replication and protects against symptomatic disease makes this species ideally suited to investigate the immune response primed by preconceptual infection. Using this model where susceptibility to ZIKV can be temporally controlled by delayed administration of type I IFN receptor blocking antibody, we show even asymptomatic primary infection protects against re-infection, and that these protective benefits extend to re-infection during allogenic pregnancy.

Importantly, ZIKV primary infection in wildtype mice used to model human infections that are mostly subclinical and primarily associated with only transient self-resolving viremia does not replicate all aspects of human infection. For example, ZIKV infection in non-pregnant individuals remains asymptomatic despite presumed functional neutralization of type I IFN responsiveness though STAT2 inactivation [10, 11]. Thus, the enhanced vulnerability of type I IFN receptor deficient mice that almost uniformly develop symptomatic and often fatal infection [34, 70], suggests STAT2-indepenent, type I IFN-dependent cell activation pathways may also be important in protection against ZIKV symptomatic infection. Potential candidates are type I IFN induced STAT3 activation that overrides the pro-inflammatory effects of activated STAT1 and STAT2 in myeloid cells [71], or STAT5 phosphorylation that drives CD4^+^ T cell differentiation into FOXP3^+^ regulatory cells [72]. Nonetheless, despite this potential limitation regarding how asymptomatic primary infection is achieved, protective immunity efficiently primed by abortive infection we demonstrate in wildtype mice further highlights the immunogenicity of endogenous viral antigens recently shown with minimally-replicating live-attenuated viral strains, non-replicating inactivated virus or nucleic acid-based candidate vaccine formulations [13–18, 20, 21].

In the broader epidemiological context, antibody-mediated protection against re-infection during pregnancy primed by preconceptual ZIKV primary infection has important translational implications for new therapeutic strategies aimed at identifying at-risk individuals, and protecting expecting mothers and their fetal offspring. Considering an estimated attack rate that exceeds 90% [44], together with an ~80% rate of subclinical infection among individuals with newly acquired infection [11, 12], a majority of reproductive age women in ZIKV endemic areas likely have naturally acquired immunity against re-infection primed by resolved prior infections. Thus, despite very promising protective benefits having been recently shown for several ZIKV candidate vaccines in preclinical infection models involving non-pregnant animals or mice during syngeneic pregnancies [13–21], neither the safety of these formulations, nor their protective efficacy during pregnancies that recapitulate the heterogeneity between maternal and fetal expressed antigens representative of naturally outbred human populations have been established. Thus, our data highlighting that protection against re-infection primed by preconceptual infection are functionally retained during allogeneic pregnancy adds an important, but previously unaddressed perspective on how protection against prenatal infection can be achieved.

Our finding that primary abortive infection primes robust expansion of anti-ZIKV neutralizing antibodies that functionally persist during pregnancy and can be found amongst individual concepti proportional to their degree of protection, together with recent studies showing donor serum or purified human monoclonal antibodies can transfer protection against ZIKV infection in pregnant hosts [43, 47], points to serological screening for viral neutralizing antibodies as a practical approach for distinguishing susceptible at-risk individuals from those with naturally acquired immunity. Considering reduced placental transfer of maternal antibodies in mice compared with other mammalian species [73, 74], the 10^2^ to 10^4^-fold reduced ZIKV levels we demonstrate in individual mouse concepti likely underestimates the degree of protection achievable for human fetal offspring. In turn, the inverse correlation between ZIKV RNA levels amongst individual concepti following secondary infection and levels of anti-ZIKV IgG antibody suggest individual fetal offspring are near the threshold for minimal amount of passively transferred maternal antibody required for protection against congenital invasion in this model using mice rendered susceptible with type I IFN receptor blockade. Taken together, these results showing cross-lineage immunity against ZIKV prenatal infection conferred by primary abortive infection, together with protection primed by minimally replicating, attenuated ZIKV vaccine candidates [14, 20, 21], underscores the therapeutic potential of preconceptual strategies that prime accumulation of high titer neutralizing antibodies for broadly protecting susceptible reproductive age women. On the other hand, since primary ZIKV infection during early pregnancy in mice also primes the accumulation of neutralizing antibodies that are presumably similarly protective [49], averting congenital fetal invasion may require increased functional thresholds based on antibody levels and/or affinity to viral expressed antigens. Thus, important next-steps are to further investigate the degree of protection against ZIKV prenatal infection primed by preconceptual primary infection, and whether the reduced virus levels we find in maternal tissues are below the threshold required for pathological fetal infection in animals where gestational length, and placental expression of factors that influence antibody function (e.g. neonatal Fc receptor, complement regulatory protein Crry), are more representative of human pregnancy [73–75].

## Materials and methods

### Ethics statement

Experiments involving animals were performed under Cincinnati Children’s Hospital Institutional Animal Care and Use Committee (IACUC) approved protocols (Assurance Number 2013–0170). These protocols strictly adhere to recommendations described in the National Research Council’s “Guide for the Care and Use of Laboratory Animals” and American Veterinary Medical Association's "Report of the AVMA Panel on Euthanasia”.

### Mouse experiments

C57BL/6 (H-2^b^) and Balb/c (H-2^d^) mice were purchased from the National Cancer Institute Charles River Laboratories (Frederick, Maryland), and maintained under specific-pathogen free conditions at the Cincinnati Children’s Hospital. For all Zika challenge studies, 6–8 week old, sex-matched C57BL/6 mice were randomly assigned to experimental groups. For experiments during gestation, 6–8 week old Balb/c males were used to sire allogeneic pregnancies in C57BL/6 females.

### Virus and cells

Zika virus (ZIKV) strains PRVABC59 (Puerto Rico, 2015) and MR766 (Uganda, 1947) were obtained from the US Center for Disease and Prevention (Atlanta, Georgia) and American Type Culture Collection (Manassas, Virginia), respectively [50]. Virus stocks were propagated and titred based on the number of plaque forming units (PFUs) in semi-confluent monolayers of Vero cells (American Type Culture Collection; Manassas, Virginia). All experiments were carried out under biosafety level 2 (BSL2) containment at Cincinnati Children’s Hospital.

### ZIKV infection

For abortive infections, mice were inoculated subcutaneously in the lateral flank with 10^6^ PFU ZIKV suspended in 100 μL sterile saline. For challenge studies, non-pregnant or allogeneic pregnant females (embryonic day 10.5) with and without primary abortive infection were administered 1 mg anti-type I IFN receptor antibody (MAR1-5A3; BE0241; BioXcell, West Lebanon, New Hampshire) intraperitoneally 24 hours prior to and on the day of infection, and were subsequently boosted (0.5 mg/dose) every five days thereafter. Mice were checked daily and assigned the following clinical disease score (1 healthy; 2 limited ruffled fur; 3 ruffled fur throughout; 4 mild lethargy; 5 limited movement; 6 moribund or uncontrolled spastic movements; 7 deceased) as previously described [76]. For serum harvest, blood from donor mice was obtained 21 days after initial infection, spun, filtered, and stored at -20°C. For heat inactivation, serum was incubated at 56°C for 30 minutes. For adoptive transfer, 300 μl serum (representing ~1/3 serum volume per donor animal) was administered i.p. to each recipient mouse one day prior to ZIKV infection. For Fcγ receptor neutralization *in vivo*, 250 μg of anti-CD16/CD32 antibody (clone 2.4G2, BioXcell) were administered intraperitoneally to mice day -1, 0 and 2 relative to ZIKV infection as described [45, 46]. For cell depletion, anti-CD4 (GK1.5; BE0003-1; BioXcell) and/or anti-CD8 (2.43; BE0061; BioXcell) antibodies (500 μg/mouse) were administered intraperitoneally to mice as previously described [68] one day prior ZIKV infection.

### Measurement of viral burden

Following ZIKV infection, individual tissues were harvested and homogenized, whereas serum was collected after coagulation and centrifugation. Homogenized tissue and serum samples were extracted with the RNeasy mini kit (Qiagen, Hilden, Germany), and levels of ZIKV RNA evaluated by TaqMan (ThermoFisher, Waltham, Massachusetts) one-step quantitative reverse transcriptase PCR (qRT-PCR) on an ABI 7500 Fast instrument, using a previously described primer/probe set: forward primer, 5’-CCGCTGCCCAACACAAG-3’; reverse primer, 5’-CCACTAACGTTCTTTTGCAGACAT-3’; probe 5’/FAM/AGCCTACCTTGACAAGCAATCAGACACTCAA/NFQ-MGB/-3’ [34, 77]. Viral burden for each entire tissue was calculated by interpolation from a standard curve produced using serial 10-fold dilutions of ZIKV, and expressed on a log_10_ scale as number of viral copies.

### Quantification of ZIKV-specific antibodies

Blood was collected from infected mice compared with uninfected controls and serum was isolated after coagulation and centrifugation. Each individual concepti (fetus plus placenta) was collected 3 days after prenatal infection from primed or naive pregnant females, homogenized in PBS (1 mL) and stored at -80°C. For ELISA, 96-well plates were coated overnight with purified ZIKV ENV (MBS319787) or NS1 (MBS319788) proteins, blocked with BSA (1%), incubated with serial dilutions of serum (starting at 1:10 dilution) or clarified fetal tissue homogenates (1:4 dilution). ZIKV-specific antibodies were probed with biotinylated secondary antibodies including rat anti-mouse IgA (clone 11-44-2; 13-5994-82; ThermoFisher), IgM (clone eB121-15F9; 13-5890-85; ThermoFisher), IgG1 (clone A85-1; 553441; BD Bioscience, San Jose, California), IgG2a (clone R10-15; 553388; BD Bioscience), IgG2b (clone R12-3; 553393; BD Bioscience), IgG3 (clone R40-82; 553401; BD Pharmingen), developed with streptavidin-peroxidase (554066; BD Bioscience) using o-phenylenediamine dihydrochloride as a substrate and reading absorbance at 450 nm (A_450_).

### Virus neutralization assays

For viral neutralization, serial dilutions of the serum from mice with and without primary abortive infection were pre-incubated with 10^2^ PFU ZIKV PRVABC59 or MR766 for 1 hour at 37°C. To investigate functionally neutralizing antibodies in concepti, clarified fetal homogenate was UV treated for 1 hour to inactivate any residual virus [78], and subsequently incubated with 10^2^ PFU ZIKV PRVABC59 at a 1:100 dilution for 1 hour at 37°C. Following incubation, protection against plaque formation by each virus-antibody complex was assessed in Vero cell monolayers by first incubation at 37°C for 1 hour, followed by overlaying monolayer cells in each well with methyl cellulose (1%), and enumeration of plaques 72 hours thereafter as described [43].

### Quantification and statistical analysis

All data were analyzed using GraphPad Prism software. For viral burden, levels of ZIKV RNA within each individual data set, were analyzed using the non-parametric Mann-Whitney test (two groups) or ANOVA (3 or more experimental groups). Linear regression was performed to determine correlation between ZIKA RNA and ZIKA-specific IgG levels in fetal tissue homogenates. *P* < 0.05 was taken as statistical significance.

## Supporting information

S1 Fig Protection against ZIKV re-infection primed by primary asymptomatic infection is associated with neutralizing serum that is resistant to heat-inactivation and *in vivo* T cell depletion. (**A**) Percent reduction in ZIKV plaques after pre-incubation with each dilution of fresh serum from mice day 21 after asymptomatic primary infection compared with serum from the same animal incubated at 56°C for 30 minutes. (**B**) Schematic illustrating when anti-CD4 and/or anti-CD8 depleting antibodies are administered relative to asymptomatic ZIKV primary infection and secondary ZIKV challenge; and ZIKV genome copies in the serum and each tissue day 3 after infection for each group of mice. Each point depicts the data from an individual mouse that is representative of at least three independent experiments each with similar results. Bar, mean ± one standard error; LOD, limits of detection; *** *p* < 0.001.
(TIF)

## References

[1] Fauci AS, Morens DM. Zika Virus in the Americas—Yet Another Arbovirus Threat. N Engl J Med. 2016;374(7):601–4. doi: 10.1056/NEJMp1600297 . 26761185 10.1056/NEJMp1600297

[2] Lazear HM, Diamond MS. Zika Virus: New Clinical Syndromes and Its Emergence in the Western Hemisphere. J Virol. 2016;90(10):4864–75. doi: 10.1128/JVI.00252-16 26962217 10.1128/JVI.00252-16 PMC4859708

[3] Coyne CB, Lazear HM. Zika virus—reigniting the TORCH. Nat Rev Microbiol. 2016;14(11):707–15. doi: 10.1038/nrmicro.2016.125 . 27573577 10.1038/nrmicro.2016.125

[4] Miner JJ, Diamond MS. Zika Virus Pathogenesis and Tissue Tropism. Cell Host Microbe. 2017;21(2):134–42. doi: 10.1016/j.chom.2017.01.004 28182948 10.1016/j.chom.2017.01.004 PMC5328190

[5] Brasil P, Pereira JP Jr., Moreira ME, Ribeiro Nogueira RM, Damasceno L, Wakimoto M, et al Zika Virus Infection in Pregnant Women in Rio de Janeiro. N Engl J Med. 2016;375(24):2321–34. doi: 10.1056/NEJMoa1602412 . 26943629 10.1056/NEJMoa1602412 PMC5323261

[6] Martines RB, Bhatnagar J, Keating MK, Silva-Flannery L, Muehlenbachs A, Gary J, et al Notes from the Field: Evidence of Zika Virus Infection in Brain and Placental Tissues from Two Congenitally Infected Newborns and Two Fetal Losses—Brazil, 2015. MMWR Morb Mortal Wkly Rep. 2016;65(6):159–60. doi: 10.15585/mmwr.mm6506e1 . 26890059 10.15585/mmwr.mm6506e1

[7] Mlakar J, Korva M, Tul N, Popovic M, Poljsak-Prijatelj M, Mraz J, et al Zika Virus Associated with Microcephaly. N Engl J Med. 2016;374(10):951–8. doi: 10.1056/NEJMoa1600651 . 26862926 10.1056/NEJMoa1600651

[8] Ventura CV, Maia M, Bravo-Filho V, Gois AL, Belfort R Jr. Zika virus in Brazil and macular atrophy in a child with microcephaly. Lancet. 2016;387(10015):228 doi: 10.1016/S0140-6736(16)00006-4 . 26775125 10.1016/S0140-6736(16)00006-4

[9] Pacheco O, Beltran M, Nelson CA, Valencia D, Tolosa N, Farr SL, et al Zika Virus Disease in Colombia—Preliminary Report. N Engl J Med. 2016 doi: 10.1056/NEJMoa1604037 . 27305043 10.1056/NEJMoa1604037

[10] Musso D, Gubler DJ. Zika Virus. Clin Microbiol Rev. 2016;29(3):487–524. doi: 10.1128/CMR.00072-15 27029595 10.1128/CMR.00072-15 PMC4861986

[11] Duffy MR, Chen TH, Hancock WT, Powers AM, Kool JL, Lanciotti RS, et al Zika virus outbreak on Yap Island, Federated States of Micronesia. N Engl J Med. 2009;360(24):2536–43. doi: 10.1056/NEJMoa0805715 . 19516034 10.1056/NEJMoa0805715

[12] Aubry M, Teissier A, Huart M, Merceron S, Vanhomwegen J, Roche C, et al Zika Virus Seroprevalence, French Polynesia, 2014–2015. Emerg Infect Dis. 2017;23(4). doi: 10.3201/eid2304.161549 . 28084987 10.3201/eid2304.161549 PMC5367400

[13] Abbink P, Larocca RA, De La Barrera RA, Bricault CA, Moseley ET, Boyd M, et al Protective efficacy of multiple vaccine platforms against Zika virus challenge in rhesus monkeys. Science. 2016 doi: 10.1126/science.aah6157 . 27492477 10.1126/science.aah6157 PMC5237380

[14] Shan C, Muruato AE, Nunes BTD, Luo H, Xie X, Medeiros DBA, et al A live-attenuated Zika virus vaccine candidate induces sterilizing immunity in mouse models. Nat Med. 2017 doi: 10.1038/nm.4322 . 28394328 10.1038/nm.4322 PMC6276361

[15] Larocca RA, Abbink P, Peron JP, Zanotto PM, Iampietro MJ, Badamchi-Zadeh A, et al Vaccine protection against Zika virus from Brazil. Nature. 2016;536(7617):474–8. doi: 10.1038/nature18952 27355570 10.1038/nature18952 PMC5003703

[16] Pardi N, Hogan MJ, Pelc RS, Muramatsu H, Andersen H, DeMaso CR, et al Zika virus protection by a single low-dose nucleoside-modified mRNA vaccination. Nature. 2017 doi: 10.1038/nature21428 . 28151488 10.1038/nature21428 PMC5344708

[17] Richner JM, Himansu S, Dowd KA, Butler SL, Salazar V, Fox JM, et al Modified mRNA Vaccines Protect against Zika Virus Infection. Cell. 2017 doi: 10.1016/j.cell.2017.02.017 . 28340344 10.1016/j.cell.2017.03.016

[18] Dowd KA, Ko SY, Morabito KM, Yang ES, Pelc RS, DeMaso CR, et al Rapid development of a DNA vaccine for Zika virus. Science. 2016;354(6309):237–40. doi: 10.1126/science.aai9137 . 27708058 10.1126/science.aai9137 PMC5304212

[19] Sumathy K, Kulkarni B, Gondu RK, Ponnuru SK, Bonguram N, Eligeti R, et al Protective efficacy of Zika vaccine in AG129 mouse model. Sci Rep. 2017;7:46375 Epub 2017/04/13. doi: 10.1038/srep46375 28401907 10.1038/srep46375 PMC5388871

[20] Richner JM, Jagger BW, Shan C, Fontes CR, Dowd KA, Cao B, et al Vaccine Mediated Protection Against Zika Virus-Induced Congenital Disease. Cell. 2017;170(2):273–83 e12. doi: 10.1016/j.cell.2017.06.040 28708997 10.1016/j.cell.2017.06.040 PMC5546158

[21] Shan C, Muruato AE, Jagger BW, Richner J, Nunes BTD, Medeiros DBA, et al A single-dose live-attenuated vaccine prevents Zika virus pregnancy transmission and testis damage. Nat Commun. 2017;8(1):676 doi: 10.1038/s41467-017-00737-8 28939807 10.1038/s41467-017-00737-8 PMC5610254

[22] Boue A, Nicolas A, Montagnon B. Reinfection with rubella in pregnant women. Lancet. 1971;1(7712):1251–3. . 4104713 10.1016/s0140-6736(71)91775-2

[23] Harcourt GC, Best JM, Banatvala JE. Rubella-specific serum and nasopharyngeal antibodies in volunteers with naturally acquired and vaccine-induced immunity after intranasal challenge. J Infect Dis. 1980;142(2):145–55. . 7410897 10.1093/infdis/142.2.145

[24] Lamont RF, Sobel JD, Carrington D, Mazaki-Tovi S, Kusanovic JP, Vaisbuch E, et al Varicella-zoster virus (chickenpox) infection in pregnancy. BJOG. 2011;118(10):1155–62. doi: 10.1111/j.1471-0528.2011.02983.x 21585641 10.1111/j.1471-0528.2011.02983.x PMC3155623

[25] Boppana SB, Rivera LB, Fowler KB, Mach M, Britt WJ. Intrauterine transmission of cytomegalovirus to infants of women with preconceptional immunity. N Engl J Med. 2001;344(18):1366–71. doi: 10.1056/NEJM200105033441804 . 11333993 10.1056/NEJM200105033441804

[26] Siston AM, Rasmussen SA, Honein MA, Fry AM, Seib K, Callaghan WM, et al Pandemic 2009 influenza A(H1N1) virus illness among pregnant women in the United States. JAMA. 2010;303(15):1517–25. doi: 10.1001/jama.2010.479 . 20407061 10.1001/jama.2010.479 PMC5823273

[27] Clark DR, Chaturvedi V, Kinder JM, Jiang TT, Xin L, Ertelt JM, et al Perinatal Listeria monocytogenes susceptibility despite preconceptual priming and maintenance of pathogen-specific CD8(+) T cells during pregnancy. Cell Mol Immunol. 2014;11(6):595–605. Epub 2014/09/23. doi: 10.1038/cmi.2014.84 25242275 10.1038/cmi.2014.84 PMC4220843

[28] McGregor IA. Epidemiology, malaria and pregnancy. Am J Trop Med Hyg. 1984;33(4):517–25. . 6383091 10.4269/ajtmh.1984.33.517

[29] Rogerson SJ. Malaria in pregnancy and the newborn. Adv Exp Med Biol. 2010;659:139–52. doi: 10.1007/978-1-4419-0981-7_12 . 20204762 10.1007/978-1-4419-0981-7_12

[30] Hammon WM, Sather GE. Immunity of hamsters to West Nile and Murray Valley viruses following immunization with St. Louis and Japanese B. Proc Soc Exp Biol Med. 1956;91(3):521–4. . 13322987 10.3181/00379727-91-22314

[31] Lobigs M, Diamond MS. Feasibility of cross-protective vaccination against flaviviruses of the Japanese encephalitis serocomplex. Expert Rev Vaccines. 2012;11(2):177–87. doi: 10.1586/erv.11.180 22309667 10.1586/erv.11.180 PMC3337329

[32] Price WH, Thind IS, O'Leary W, el Dadah AH. A protective mechanism induced by live group B arboviruses against heterologous group B arboviruses independent of serum neutralizing antibodies or interferon. Am J Epidemiol. 1967;86(1):11–27. . 4378109 10.1093/oxfordjournals.aje.a120716

[33] O'Leary DR, Kuhn S, Kniss KL, Hinckley AF, Rasmussen SA, Pape WJ, et al Birth outcomes following West Nile Virus infection of pregnant women in the United States: 2003–2004. Pediatrics. 2006;117(3):e537–45. doi: 10.1542/peds.2005-2024 . 16510632 10.1542/peds.2005-2024

[34] Lazear HM, Govero J, Smith AM, Platt DJ, Fernandez E, Miner JJ, et al A Mouse Model of Zika Virus Pathogenesis. Cell Host Microbe. 2016;19(5):720–30. doi: 10.1016/j.chom.2016.03.010 27066744 10.1016/j.chom.2016.03.010 PMC4866885

[35] Miner JJ, Cao B, Govero J, Smith AM, Fernandez E, Cabrera OH, et al Zika Virus Infection during Pregnancy in Mice Causes Placental Damage and Fetal Demise. Cell. 2016;165(5):1081–91. doi: 10.1016/j.cell.2016.05.008 27180225 10.1016/j.cell.2016.05.008 PMC4874881

[36] Grant A, Ponia SS, Tripathi S, Balasubramaniam V, Miorin L, Sourisseau M, et al Zika Virus Targets Human STAT2 to Inhibit Type I Interferon Signaling. Cell Host Microbe. 2016;19(6):882–90. doi: 10.1016/j.chom.2016.05.009 27212660 10.1016/j.chom.2016.05.009 PMC4900918

[37] Kumar A, Hou S, Airo AM, Limonta D, Mancinelli V, Branton W, et al Zika virus inhibits type-I interferon production and downstream signaling. EMBO Rep. 2016;17(12):1766–75. doi: 10.15252/embr.201642627 27797853 10.15252/embr.201642627 PMC5283583

[38] Aliota MT, Dudley DM, Newman CM, Mohr EL, Gellerup DD, Breitbach ME, et al Heterologous Protection against Asian Zika Virus Challenge in Rhesus Macaques. PLoS Negl Trop Dis. 2016;10(12):e0005168 doi: 10.1371/journal.pntd.0005168 27911897 10.1371/journal.pntd.0005168 PMC5135040

[39] Dudley DM, Aliota MT, Mohr EL, Weiler AM, Lehrer-Brey G, Weisgrau KL, et al A rhesus macaque model of Asian-lineage Zika virus infection. Nat Commun. 2016;7:12204 doi: 10.1038/ncomms12204 27352279 10.1038/ncomms12204 PMC4931337

[40] Osuna CE, Lim SY, Deleage C, Griffin BD, Stein D, Schroeder LT, et al Zika viral dynamics and shedding in rhesus and cynomolgus macaques. Nat Med. 2016;22(12):1448–55. doi: 10.1038/nm.4206 . 27694931 10.1038/nm.4206 PMC5293594

[41] Elong Ngono A, Vizcarra EA, Tang WW, Sheets N, Joo Y, Kim K, et al Mapping and Role of the CD8+ T Cell Response During Primary Zika Virus Infection in Mice. Cell Host Microbe. 2017;21(1):35–46. doi: 10.1016/j.chom.2016.12.010 28081442 10.1016/j.chom.2016.12.010 PMC5234855

[42] Huang H, Li S, Zhang Y, Han X, Jia B, Liu H, et al CD8+ T Cell Immune Response in Immunocompetent Mice during Zika Virus Infection. J Virol. 2017 doi: 10.1128/JVI.00900-17 . 28835502 10.1128/JVI.00900-17 PMC5660488

[43] Sapparapu G, Fernandez E, Kose N, Bin C, Fox JM, Bombardi RG, et al Neutralizing human antibodies prevent Zika virus replication and fetal disease in mice. Nature. 2016;540(7633):443–7. doi: 10.1038/nature20564 . 27819683 10.1038/nature20564 PMC5583716

[44] Kucharski AJ, Funk S, Eggo RM, Mallet HP, Edmunds WJ, Nilles EJ. Transmission Dynamics of Zika Virus in Island Populations: A Modelling Analysis of the 2013–14 French Polynesia Outbreak. PLoS Negl Trop Dis. 2016;10(5):e0004726 doi: 10.1371/journal.pntd.0004726 27186984 10.1371/journal.pntd.0004726 PMC4871342

[45] Araujo-Jorge T, Rivera MT, el Bouhdidi A, Daeron M, Carlier Y. An Fc gamma RII-, Fc gamma RIII-specific monoclonal antibody (2.4G2) decreases acute Trypanosoma cruzi infection in mice. Infect Immun. 1993;61(11):4925–8. Epub 1993/11/01. 8406898 10.1128/iai.61.11.4925-4928.1993 PMC281258

[46] Kurlander RJ, Ellison DM, Hall J. The blockade of Fc receptor-mediated clearance of immune complexes in vivo by a monoclonal antibody (2.4G2) directed against Fc receptors on murine leukocytes. J Immunol. 1984;133(2):855–62. Epub 1984/08/01. . 6736648

[47] Wang S, Hong S, Deng YQ, Ye Q, Zhao LZ, Zhang FC, et al Transfer of convalescent serum to pregnant mice prevents Zika virus infection and microcephaly in offspring. Cell Res. 2017;27(1):158–60. doi: 10.1038/cr.2016.144 27922617 10.1038/cr.2016.144 PMC5223229

[48] Pardy RD, Rajah MM, Condotta SA, Taylor NG, Sagan SM, Richer MJ. Analysis of the T Cell Response to Zika Virus and Identification of a Novel CD8+ T Cell Epitope in Immunocompetent Mice. PLoS Pathog. 2017;13(2):e1006184 doi: 10.1371/journal.ppat.1006184 . 28231312 10.1371/journal.ppat.1006184 PMC5322871

[49] Winkler CW, Myers LM, Woods TA, Messer RJ, Carmody AB, McNally KL, et al Adaptive Immune Responses to Zika Virus Are Important for Controlling Virus Infection and Preventing Infection in Brain and Testes. J Immunol. 2017;198(9):3526–35. doi: 10.4049/jimmunol.1601949 . 28330900 10.4049/jimmunol.1601949 PMC5701572

[50] Lanciotti RS, Lambert AJ, Holodniy M, Saavedra S, Signor Ldel C. Phylogeny of Zika Virus in Western Hemisphere, 2015. Emerg Infect Dis. 2016;22(5):933–5. doi: 10.3201/eid2205.160065 27088323 10.3201/eid2205.160065 PMC4861537

[51] Halstead SB. Pathogenesis of dengue: challenges to molecular biology. Science. 1988;239(4839):476–81. . 3277268 10.1126/science.3277268

[52] Halstead SB. Dengue. Lancet. 2007;370(9599):1644–52. doi: 10.1016/S0140-6736(07)61687-0 . 17993365 10.1016/S0140-6736(07)61687-0

[53] Sabin AB. Research on dengue during World War II. Am J Trop Med Hyg. 1952;1(1):30–50. . 14903434 10.4269/ajtmh.1952.1.30

[54] Dowd KA, DeMaso CR, Pelc RS, Speer SD, Smith AR, Goo L, et al Broadly Neutralizing Activity of Zika Virus-Immune Sera Identifies a Single Viral Serotype. Cell Rep. 2016;16(6):1485–91. doi: 10.1016/j.celrep.2016.07.049 27481466 10.1016/j.celrep.2016.07.049 PMC5004740

[55] Erlebacher A. Immunology of the maternal-fetal interface. Annu Rev Immunol. 2013;31:387–411. Epub 2013/01/10. doi: 10.1146/annurev-immunol-032712-100003 . 23298207 10.1146/annurev-immunol-032712-100003

[56] Erlebacher A. Mechanisms of T cell tolerance towards the allogeneic fetus. Nat Rev Immunol. 2013;13(1):23–33. doi: 10.1038/nri3361 . 23237963 10.1038/nri3361

[57] Barba-Spaeth G, Dejnirattisai W, Rouvinski A, Vaney MC, Medits I, Sharma A, et al Structural basis of potent Zika-dengue virus antibody cross-neutralization. Nature. 2016;536(7614):48–53. doi: 10.1038/nature18938 . 27338953 10.1038/nature18938

[58] Stettler K, Beltramello M, Espinosa DA, Graham V, Cassotta A, Bianchi S, et al Specificity, cross-reactivity, and function of antibodies elicited by Zika virus infection. Science. 2016;353(6301):823–6. doi: 10.1126/science.aaf8505 . 27417494 10.1126/science.aaf8505

[59] Zhao H, Fernandez E, Dowd KA, Speer SD, Platt DJ, Gorman MJ, et al Structural Basis of Zika Virus-Specific Antibody Protection. Cell. 2016;166(4):1016–27. doi: 10.1016/j.cell.2016.07.020 27475895 10.1016/j.cell.2016.07.020 PMC4983199

[60] Mehlhop E, Nelson S, Jost CA, Gorlatov S, Johnson S, Fremont DH, et al Complement protein C1q reduces the stoichiometric threshold for antibody-mediated neutralization of West Nile virus. Cell Host Microbe. 2009;6(4):381–91. doi: 10.1016/j.chom.2009.09.003 19837377 10.1016/j.chom.2009.09.003 PMC2782387

[61] Nimmerjahn F, Ravetch JV. Fcgamma receptors as regulators of immune responses. Nat Rev Immunol. 2008;8(1):34–47. Epub 2007/12/08. doi: 10.1038/nri2206 . 18064051 10.1038/nri2206

[62] Sorman A, Zhang L, Ding Z, Heyman B. How antibodies use complement to regulate antibody responses. Mol Immunol. 2014;61(2):79–88. Epub 2014/07/09. doi: 10.1016/j.molimm.2014.06.010 . 25001046 10.1016/j.molimm.2014.06.010

[63] George J, Valiant WG, Mattapallil MJ, Walker M, Huang YS, Vanlandingham DL, et al Prior Exposure to Zika Virus Significantly Enhances Peak Dengue-2 Viremia in Rhesus Macaques. Sci Rep. 2017;7(1):10498 Epub 2017/09/07. doi: 10.1038/s41598-017-10901-1 28874759 10.1038/s41598-017-10901-1 PMC5585353

[64] Kawiecki AB, Christofferson RC. Zika Virus-Induced Antibody Response Enhances Dengue Virus Serotype 2 Replication In Vitro. J Infect Dis. 2016;214(9):1357–60. Epub 2016/08/16. doi: 10.1093/infdis/jiw377 . 27521359 10.1093/infdis/jiw377

[65] Schmidt A, Morales-Prieto DM, Pastuschek J, Frohlich K, Markert UR. Only humans have human placentas: molecular differences between mice and humans. J Reprod Immunol. 2015;108:65–71. doi: 10.1016/j.jri.2015.03.001 . 25817465 10.1016/j.jri.2015.03.001

[66] Rowe JH, Ertelt JM, Xin L, Way SS. Regulatory T cells and the immune pathogenesis of prenatal infection. Reproduction. 2013;146(6):R191–203. doi: 10.1530/REP-13-0262 23929902 10.1530/REP-13-0262 PMC3805746

[67] Rowe JH, Ertelt JM, Aguilera MN, Farrar MA, Way SS. Foxp3(+) regulatory T cell expansion required for sustaining pregnancy compromises host defense against prenatal bacterial pathogens. Cell Host Microbe. 2011;10(1):54–64. doi: 10.1016/j.chom.2011.06.005 21767812 10.1016/j.chom.2011.06.005 PMC3140139

[68] Chaturvedi V, Ertelt JM, Jiang TT, Kinder JM, Xin L, Owens KJ, et al CXCR3 blockade protects against Listeria monocytogenes infection-induced fetal wastage. The Journal of Clinical Investigation. 2015;125(4):1713–25. Epub 2015/03/10. doi: 10.1172/JCI78578 25751061 10.1172/JCI78578 PMC4396484

[69] Rowe JH, Ertelt JM, Xin L, Way SS. Listeria monocytogenes cytoplasmic entry induces fetal wastage by disrupting maternal FoxP3+ regulatory cell-sustained fetal tolerance. PLoS Pathog. 2012;8(8):e1002873 doi: 10.1371/journal.ppat.1002873 22916020 10.1371/journal.ppat.1002873 PMC3420962

[70] Aliota MT, Caine EA, Walker EC, Larkin KE, Camacho E, Osorio JE. Characterization of Lethal Zika Virus Infection in AG129 Mice. PLoS Negl Trop Dis. 2016;10(4):e0004682 doi: 10.1371/journal.pntd.0004682 27093158 10.1371/journal.pntd.0004682 PMC4836712

[71] Ho HH, Ivashkiv LB. Role of STAT3 in type I interferon responses. Negative regulation of STAT1-dependent inflammatory gene activation. J Biol Chem. 2006;281(20):14111–8. Epub 2006/03/31. doi: 10.1074/jbc.M511797200 . 16571725 10.1074/jbc.M511797200

[72] Mahmud SA, Manlove LS, Farrar MA. Interleukin-2 and STAT5 in regulatory T cell development and function. JAKSTAT. 2013;2(1):e23154 Epub 2013/09/24. doi: 10.4161/jkst.23154 24058794 10.4161/jkst.23154 PMC3670270

[73] Kim J, Mohanty S, Ganesan LP, Hua K, Jarjoura D, Hayton WL, et al FcRn in the yolk sac endoderm of mouse is required for IgG transport to fetus. J Immunol. 2009;182(5):2583–9. doi: 10.4049/jimmunol.0803247 19234152 10.4049/jimmunol.0803247 PMC2676880

[74] Pentsuk N, van der Laan JW. An interspecies comparison of placental antibody transfer: new insights into developmental toxicity testing of monoclonal antibodies. Birth Defects Res B Dev Reprod Toxicol. 2009;86(4):328–44. doi: 10.1002/bdrb.20201 . 19626656 10.1002/bdrb.20201

[75] Xu C, Mao D, Holers V, Palanca B, Cheng A, Molina H. A critical role for murine complement regulatory Crry in fetomaternal tolerance. Science. 2000;287:498–501. 10642554 10.1126/science.287.5452.498

[76] Tang WW, Young MP, Mamidi A, Regla-Nava JA, Kim K, Shresta S. A Mouse Model of Zika Virus Sexual Transmission and Vaginal Viral Replication. Cell Rep. 2016;17(12):3091–8. doi: 10.1016/j.celrep.2016.11.070 28009279 10.1016/j.celrep.2016.11.070 PMC5193244

[77] Lanciotti RS, Kosoy OL, Laven JJ, Velez JO, Lambert AJ, Johnson AJ, et al Genetic and serologic properties of Zika virus associated with an epidemic, Yap State, Micronesia, 2007. Emerg Infect Dis. 2008;14(8):1232–9. doi: 10.3201/eid1408.080287 18680646 10.3201/eid1408.080287 PMC2600394

[78] Muller JA, Harms M, Schubert A, Jansen S, Michel D, Mertens T, et al Inactivation and Environmental Stability of Zika Virus. Emerg Infect Dis. 2016;22(9):1685–7. Epub 2016/07/02. doi: 10.3201/eid2209.160664 27367466 10.3201/eid2209.160664 PMC4994368

