# Supplementary figures and images for: Preconceptual Zika virus asymptomatic infection protects against secondary prenatal infection
Source: PLoS Pathog. 2017 Nov 16;13(11):e1006684. doi: 10.1371/journal.ppat.1006684 (PMC5689831; doi:10.1371/journal.ppat.1006684)

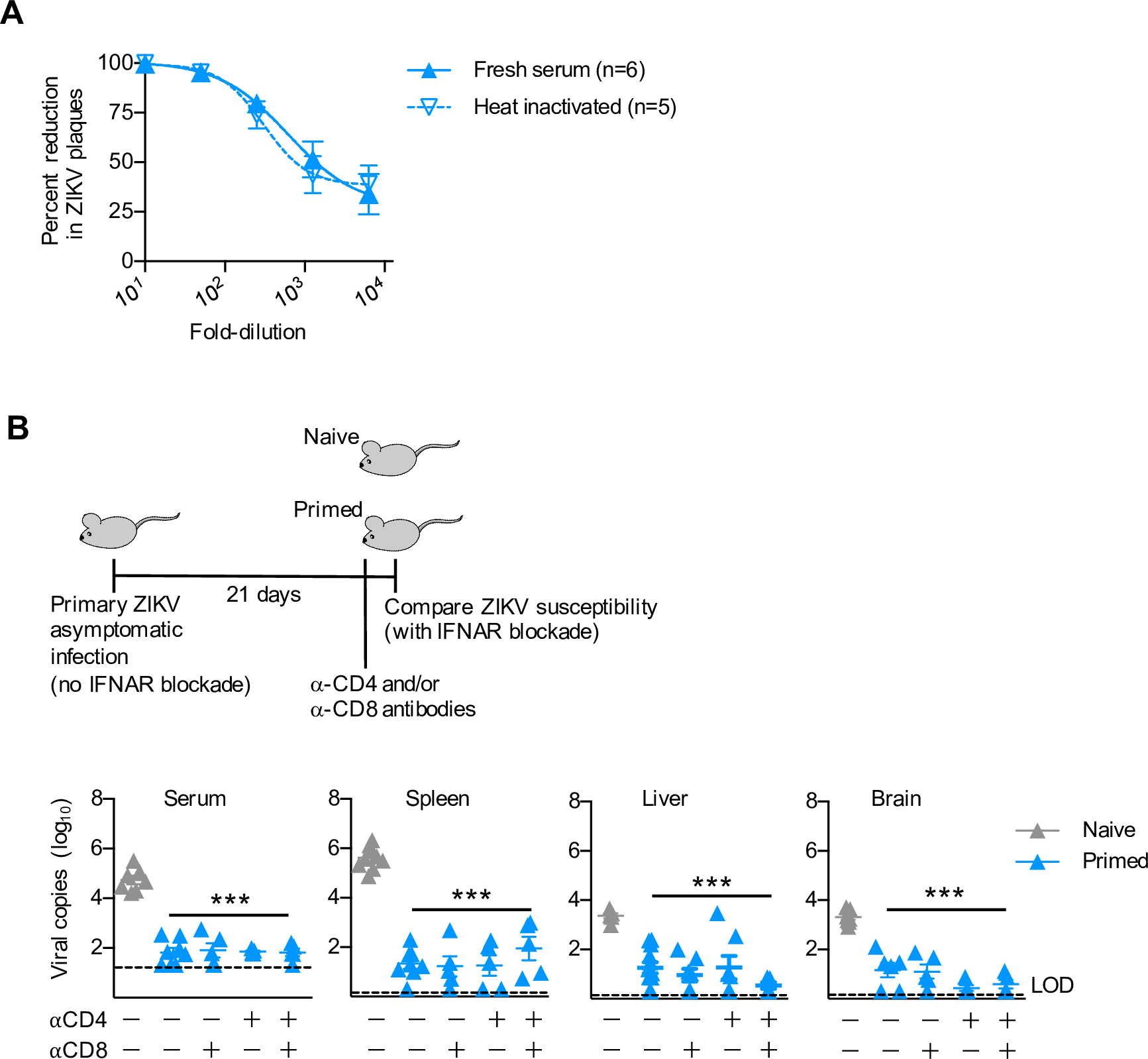

Supplement: S1 Fig — (A) Percent reduction in ZIKV plaques after pre-incubation with each dilution of fresh serum from mice day 21 after asymptomatic primary infection compared with serum from the same animal incubated at 56°C for 30 minutes. (B) Schematic illustrating when anti-CD4 and/or anti-CD8 depleting antibodies are administered relative to asymptomatic ZIKV primary infection and secondary ZIKV challenge; and ZIKV genome copies in the serum and each tissue day 3 after infection for each group of mice. Each point depicts the data from an individual mouse that is representative of at least three independent experiments each with similar results. Bar, mean ± one standard error; LOD, limits of detection; *** p < 0.001. (TIF) [file ppat.1006684.s001.tif]
